# *Achillea millefolium* Essential Oil Mitigates Peptic Ulcer in Rats through Nrf2/HO-1 Pathway

**DOI:** 10.3390/molecules27227908

**Published:** 2022-11-15

**Authors:** Manar K. Alomair, Lama S. Alabduladheem, Marwah A. Almajed, Amjad A. Alobaid, Essraa A. R. Alkhalifah, Nancy S. Younis, Maged E. Mohamed

**Affiliations:** Department of Pharmaceutical Sciences, College of Clinical Pharmacy, King Faisal University, Al-Ahsa 31982, Saudi Arabia

**Keywords:** *Achillea millefolium*, anti-inflammatory, antioxidant, apoptosis, Nrf_2_/HO-1 pathway

## Abstract

Extreme ethanol ingestion is associated with developing gastric ulcers. *Achillea millefolium* (yarrow) is one of the most commonly used herbs with numerous proven pharmacological actions. The goal of the hereby investigation is to explore the gastroprotective action of yarrow essential oil against ethanol-induced gastric ulcers and to reveal the unexplored mechanisms. Rats were distributed into five groups (*n = 6*); the control group administered 10% Tween 20, orally, for two weeks; the ethanol group administered absolute ethanol (5 mL/kg) to prompt gastric ulcer on the last day of the experiment. Yarrow essential oil 100 or 200 mg/kg + ethanol groups pretreated with yarrow oil (100 or 200 mg/kg, respectively), orally, for two weeks prior to gastric ulcer induction by absolute ethanol. Lanso + ethanol group administered 20 mg/kg lansoprazole, orally, for two weeks prior to gastric ulcer induction by ethanol. Results of the current study showed that ethanol caused several macroscopic and microscopic alterations, amplified lipid peroxidation, pro-inflammatory cytokines, and apoptotic markers, as well as diminished PGE_2_, NO, and antioxidant enzyme activities. On the other hand, animals pretreated with yarrow essential oil exhibited fewer macroscopic and microscopic modifications, reduced ulcer surface, and increased Alcian blue binding capacity, pH, and pepsin activity. In addition, yarrow essential oil groups exhibited reduced pro-inflammatory cytokines, apoptotic markers, and MDA, restored the PGE_2_ and NO levels, and recovered the antioxidant enzyme activities. Ethanol escalated Nrf2 and HO-1 expressions, whereas pretreatment of yarrow essential oil caused further intensification in Nrf2 and HO-1. To conclude, the current study suggested yarrow essential oil as a gastroprotective agent against ethanol-induced gastric lesions. This gastroprotective effect could be related to the antioxidant, anti-inflammatory, and anti-apoptotic actions of the essential oil through the instigation of the Nrf2/HO-1 pathway.

## 1. Introduction

One of the most common gastrointestinal tract diseases is the gastric ulcer [1]. These types of ulcers develop as a result of the disproportion between destructive and defensive features in the gastric mucosa [2]. The gastric ulcer that arises with alcohol or NSAID consumption is associated with physiological and/or psychological stress [3]. Ethanol is a gastric mucosal destructive factor [4] as gastric exposure to ethanol promptly stimulates neutrophils, leading to oxidative stress by intensifying the generation of reactive oxygen species (ROS) as well as the pro-inflammatory cytokines, resulting in mucosal injuries and damage. Several studies established that pro-inflammatory cytokines and oxidative homeostasis have an imperative influence on alcohol-induced gastric ulcer [5]. The nuclear factor-erythroid 2 related factor 2 (Nrf2) signaling pathway protects cells from oxidative damage by triggering endogenous antioxidant enzymes [6]. Under normal physiological conditions, Nrf2 is bound to the negative regulator Keap1, forming a complex which remains inactively in the cytoplasm or degrades by proteases [7]. Under oxidative stress conditions, Nrf2 dissociates from the cytoplasmic Keap1 complex and translocates to the nucleus causing the transcriptional initiation of phase II antioxidant enzymes’ genes, including the Heme Oxygenase-1 (HO-1) [8]. Several FDA-approved medications have been used in the management of gastric ulcers. Nevertheless, these drugs are associated with several adverse effects, including the increased risk of hospital and community-acquired pneumonia, arrhythmia, gynecomastia, and hematopoietic changes [2]; hence, the search for new compounds is essential. These new anti-gastric ulcer compounds ought to have several characteristics, including the capability to mitigate gastric inflammation and oxidative stress, as well as to boost endogenous gastric mucosa’s defensive ability.

Medicinal plants and their components have numerous biological actions. The Achillea genus comprises over a hundred species and subspecies, which are dispersed all over the world [9,10,11]. *Achillea millefolium*, family Asteraceae, widely known as “yarrow”, is a frequently used herb in the Middle East as a fresh or dried herb [12,13,14]. It is utilized in different forms, such as in tea mixtures, tablets, tinctures, and ointments, among other formulations [9,15]. This herb has been traditionally used as an astringent, antiseptic, and to manage gastrointestinal complaints, diminish menstrual contractions and inconsistency, and to stimulate wound, burn, and ulcer healing [13,16]. Commission E approved the ingestion of yarrow for appetite loss and dyspeptic disorders. Furthermore, it approved the external usage of yarrow as a sitz bath for painful, cramp-like disorders occurring in the female pelvis [9]. In the USA, yarrow is utilized as a diaphoretic constituent in the typical cold and flu formulations, and in topical styptic preparations [9]. A double-blind clinical study showed that yarrow oil exhibited an anti-inflammatory activity for skin application and prevented hypertrophic scarring in humans, proposing yarrow oil as a promising base to be used in the phytopreparations designed for the dermatological application [16]. The yarrow essential oil (YEO) exhibited several pharmacological activities such as anti-leishmanial [17], antimalarial, phytotoxic, insecticidal [18], anti-inflammatory [19], anti-hemorrhoidal [20], anti-oxidant [14], anti-microbial [21], and cytotoxic and proapoptotic properties [22]. Additionally, our laboratory formerly documented the potent protective action of YEO-regulated NF-κB and PPAR-γ pathways, which protected against dextran sulfate sodium (DSS) induced ulcerative colitis (UC) [12]. Another study suggested YEO be effective as an antitumor agent, via modulating the activity of peritoneal macrophages [23].

Yet, the protective effect of YEO against gastric ulcers is unclear. Therefore, in the current investigation, we tried to disclose the gastroprotective action of YEO against gastric ulcers caused by ethanol ingestion and reveal part of the mechanisms of action underlying.

## 2. Results

### 2.1. Effect of YEO against Ethanol-Induced Gastric Ulcers

#### 2.1.1. Macroscopic Assessment of Gastric Necrotic Damage

**Figure 1:**
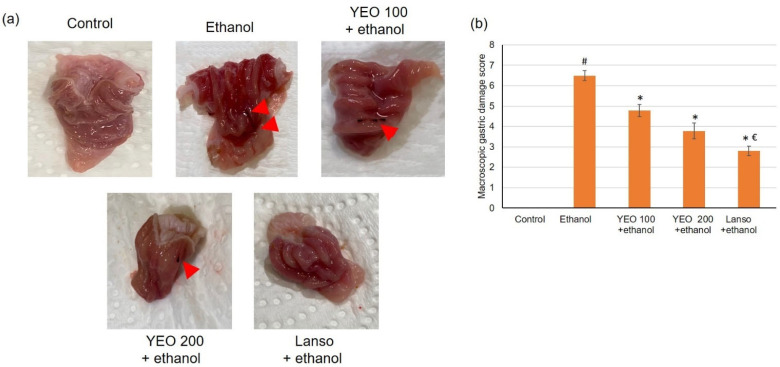
The actions of YEO (100 and 200 mg/kg) administration in acute gastric mucosal injuries induced by ethanol in rats on (**a**) demonstrative images of the separated gastric and (**b**) gastric injury scores. All data are quantified as mean ± SE, (*n = 6*). (#) defines statistically significant related to the control, (*) signifies statistically significant related to the ethanol-induced gastric mucosal injuries, and (€) designates statistically significant related to YEO 200 + ethanol via one-way ANOVA afterward Tukey’s post hoc test (*p* < 0.05).

Illustrative images of stomachs are shown in Figure 1a. The macroscopic examination of normal rats’ stomachs displayed entirely healthy pink color gastric mucosa with typical gastric mucosal thickening. The gross evaluation of ulcerated stomachs exhibited severe tissue damage as well as visible hemorrhagic mucosal lesions that presented as extended bands of hemorrhagic injuries comparable to the stomach long axis (Figure 1a, red arrowhead). Pretreatment with YEO resulted in less stomach damage as well as fewer hemorrhagic mucosal lesions. Lansoprazole (positive control) pretreated animals exhibited minor hemorrhagic vascular, but the gastric mucosa was still congested and swollen. The macroscopic gastric damage scores are shown in Figure 1b.

#### 2.1.2. Microscopic Evaluation

**Figure 2:**
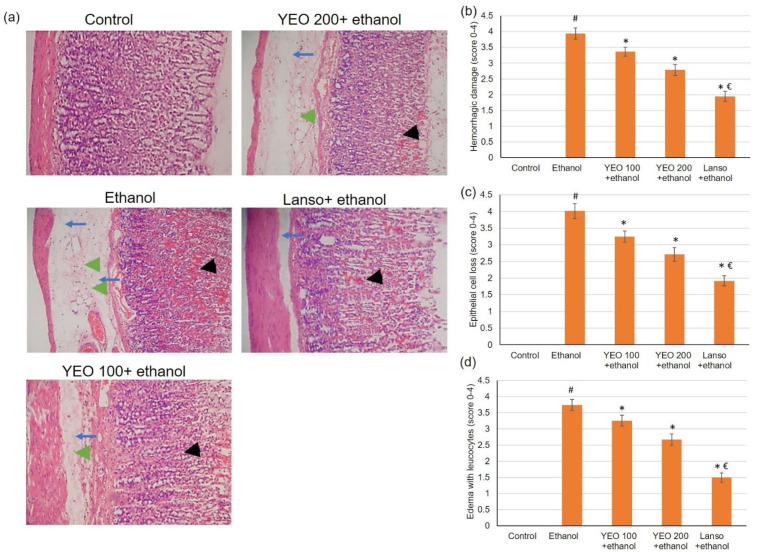
The actions of YEO (100 and 200 mg/kg) administration in acute gastric mucosal injuries induced by ethanol in rats on (**a**) histological assessment of the gastric tissue staining with H&E and the scores for (**b**) hemorrhagic injury, (**c**) epithelial cell loss, and (**d**) edema with leucocytes. Blue arrows show necrotic lesions, black arrowheads display disrupted surface epithelium and green arrowheads indicate leucocyte infiltration. (#) defines statistically significant related to the control, (*) signifies statistically significant related to the ethanol-induced gastric mucosal injuries, and (€) designates statistically significant related to YEO 200 + ethanol.

Normal animals presented intact gastric mucosa with no signs of hemorrhages, congestion, or mucosal epithelium disturbance, Figure 2a. Ethanol administration resulted in a substantial variation in the epithelium, including hemorrhagic injury (Figure 2b) and epithelial cell loss (Figure 2c), as well as edema with leucocytes (Figure 2d). Figure 2a signifies necrotic injuries infiltrated profoundly into the mucosa and expanded edema of the submucosal layer (blue arrows). Interruption of the epithelium (black arrowhead) and leucocyte infiltration (green arrowhead) were revealed in ulcerated animals. However, pretreatment with YEO and lansoprazole considerably lessened ethanol-induced epithelial destruction, contributing to the conservation of the wall structure. These macroscopic and microscopic histological investigations revealed that YEO might ameliorate ethanol-induced gastric mucosal destruction.

### 2.2. Effect of YEO on Gastric Ulcer Area and Mucus Content

Gastric ulcer surface area increased significantly in ethanol-administered animals, whereas animals pretreated with YEO and lansoprazole significantly demonstrated reduced ulcer surface area compared to the ethanol group (Table 1). Alcian blue is used as a marker of the mucus extent in the mucosa [24]. Rats with gastric injuries induced by ethanol presented a substantial reduction in Alcian blue binding ability of the mucosa (Table 1) when correlated to the control. On the contrary, pretreatment with YEO (100 or 200 mg/kg) and lansoprazole significantly amplified the Alcian blue binding ability to the mucosa compared to those of the ethanol alone group.

### 2.3. Action of YEO on the Gastric Secretion Indices

Ethanol ingestion resulted in a significant reduction in pH with a resultant escalation in gastric volume when linked to the control group. Pretreatments with YEO (100 and 200 mg/kg) produced a significant intensification in pH associated with a decline in gastric volume when related to ulcerated rats (Table 2). Concurrent ethanol administration decreased the pepsin activity of gastric juice in the ethanol-induced ulcerated animals by 2.72-fold when linked to the control, whereas pepsin activity was increased in groups pretreated with YEO (100 and 200 mg/kg) compared to the ethanol alone animals. These outcomes exposed that YEO amended ethanol-induced changes in gastric pH and volume and in the pepsin activity of gastric juice.

### 2.4. Action of Yarrow Oil on the Nrf2/HO-1 Pathway

**Figure 3:**
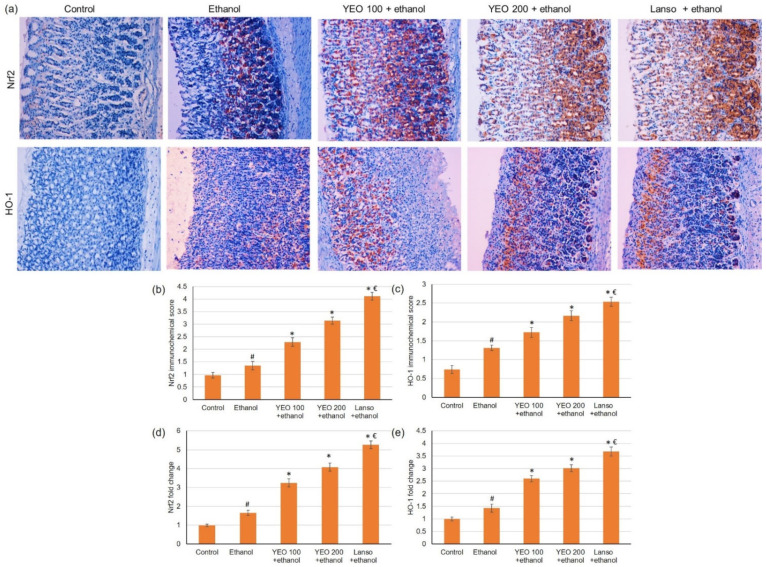
Effects of YEO (100 and 200 mg/kg) administration in acute gastric mucosal injuries induced by ethanol in rats on immunohistochemical analysis of (**a**) Nrf2 and HO-1 expressions, immunohistochemical scores for (**b**) Nrf2 and (**c**) HO1 and on the gene expression of (**d**) Nrf2 and (**e**) HO1. All data are quantified as mean ± SE, (*n = 6*). (#) defines statistically significant related to the control, (*) signifies statistically significant related to the ethanol-induced gastric mucosal injuries, and (€) designates statistically significant related to YEO 200 + ethanol via one-way ANOVA after Tukey’s post hoc test (*p* < 0.05).

Due to the imperative role of Nrf2/HO-1 signaling pathway in protecting gastric cells from oxidative damage, Nrf2 and HO-1 were evaluated using immunohistochemical and PCR. Immunohistochemical and PCR results (Figure 3) displayed that the ethanol resulted in a slightly intensified expression of Nrf2 and HO-1. However, pretreatment of YEO at the dose of 100 and 200 mg/kg dose-dependently up-regulated expressions of Nrf2 and HO-1 in ethanol-administered rats.

### 2.5. Action of YEO on Serum Inflammatory Cytokines

**Figure 4:**
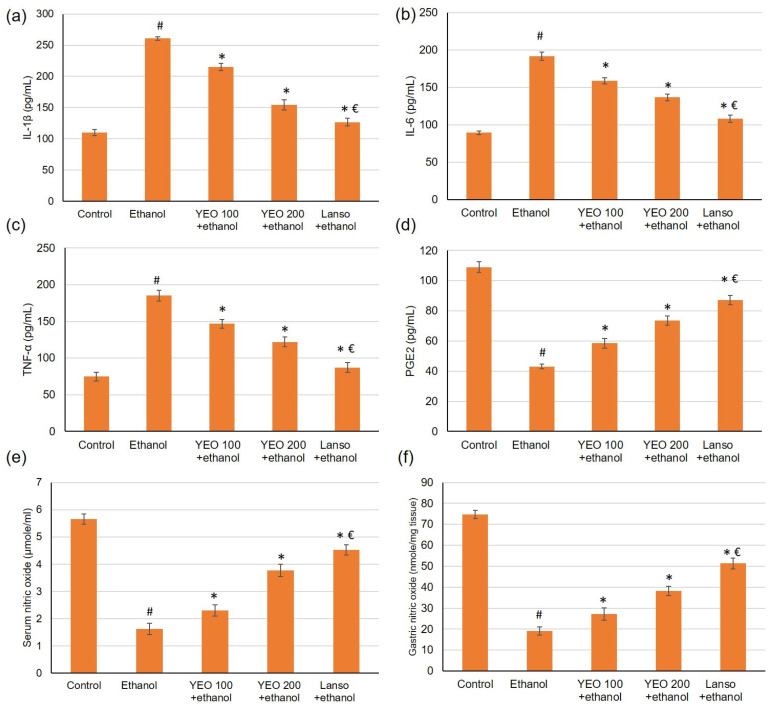
Effects of YEO (100 and 200 mg/kg) administration in acute gastric injuries induced by ethanol on serum inflammatory cytokines markers including (**a**) IL-1β, (**b**) IL-6, and (**c**) TNF-α and on gastric mucosa defensive factors including (**d**) PEG2, (**e**) serum NO, and (**f**) gastric NO. All data are quantified as mean ± SE, (*n = 6*). (#) defines statistically significant related to the control, (*) signifies statistically significant related to the ethanol-induced gastric mucosal injuries, and (€) designates statistically significant related to YEO 200 + ethanol via one-way ANOVA after Tukey’s post hoc test (*p* < 0.05).

As presented in Figure 4, ethanol considerably (*p* < 0.05) amplified the pro-inflammatory cytokines serum levels reaching a 2.37-fold increase for interleukin-1β (IL-1β), 2.1-fold rise for IL-6, and 2.47-fold upsurge for TNF-α when linked to the control group. However, these pro-inflammatory cytokines levels were markedly (*p* < 0.05) declined in the groups pretreated with lansoprazole or YEO (100 and 200 mg/kg).

### 2.6. Action of YEO on Gastric Defensive Factors

The outcomes of the current investigation disclosed that ethanol administered significantly decreased the PGE_2_ by 3.9-fold change (Figure 4d). Meanwhile, pretreatment with YEO (100 and 200 mg/kg) and lansoprazole restored the PGE_2_ level resulting in a percentage change of 35.66%, 70.48%, and 90.70%, respectively, when related to the ethanol group. As for NO, the serum and gastric levels of NO (Figure 4e,f) diminished in animals administered ethanol. Whereas the serum and gastric levels of nitric oxide amplified in the animals pretreated with YEO.

### 2.7. Action of YEO on Serum and Gastric Levels of Oxidative Stress Markers

**Figure 5:**
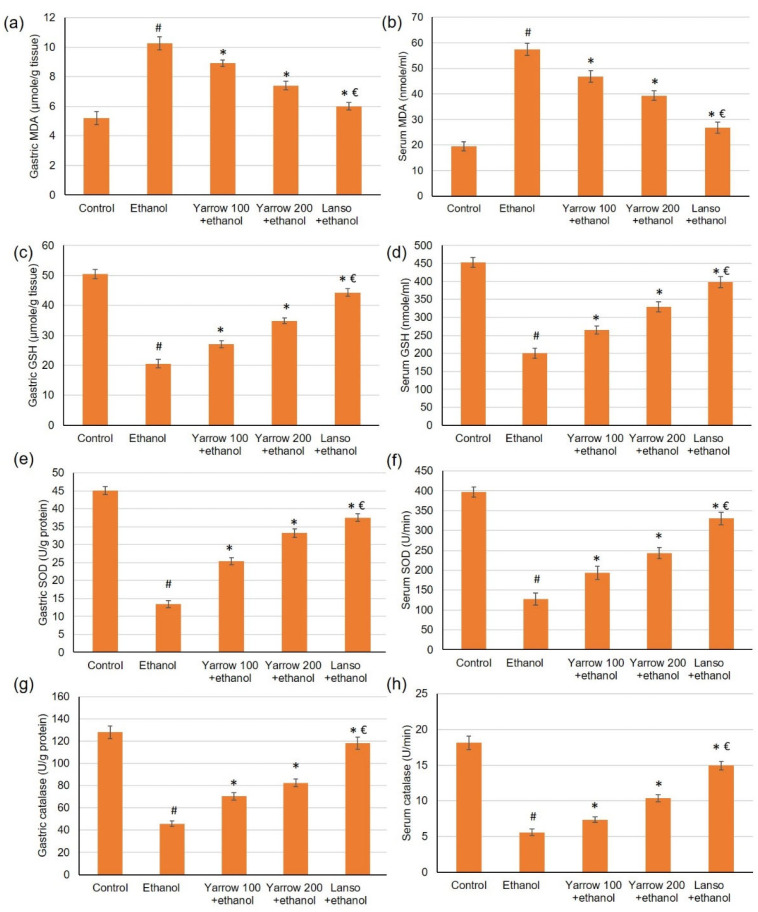
Effects of YEO (100 and 200 mg/kg) administration in acute gastric mucosal injuries induced by ethanol in rats on gastric and serum of (**a**,**b**) MDA, (**c**,**d**) GSH, (**e**,**f**) SOD and (**g**,**h**) catalase, respectively. All data are quantified as mean ± SE, (*n = 6*). (#) defines statistically significant related to the control, (*) signifies statistically significant related to the ethanol-induced gastric mucosal injuries, and (€) designates statistically significant related to YEO 200 + ethanol via one-way ANOVA afterward Tukey’s post hoc test (*p* < 0.05).

Ethanol-induced gastric injury is frequently pursued with oxidative stress as well as antioxidant enzyme depletion. Therefore, we examined one oxidative stress biomarker (MAD) and antioxidant enzyme activities (GSH, SOD, catalase), Figure 5. The results indicated that serum and gastric MDA (Figure 5a,b) were significantly elevated while GSH, SOD, and catalase activities were diminished. While the pretreatment with YEO and lansoprazole significantly lowered the serum and gastric levels of MDA. Furthermore, YEO retrieved the antioxidant enzyme GSH (Figure 5c,d), SOD (Figure 5e,f), and catalase (Figure 5g,h) activities in the gastric and serum, respectively, which were diminished by ethanol administration.

### 2.8. Effect of YEO on the Apoptosis Markers

**Figure 6:**
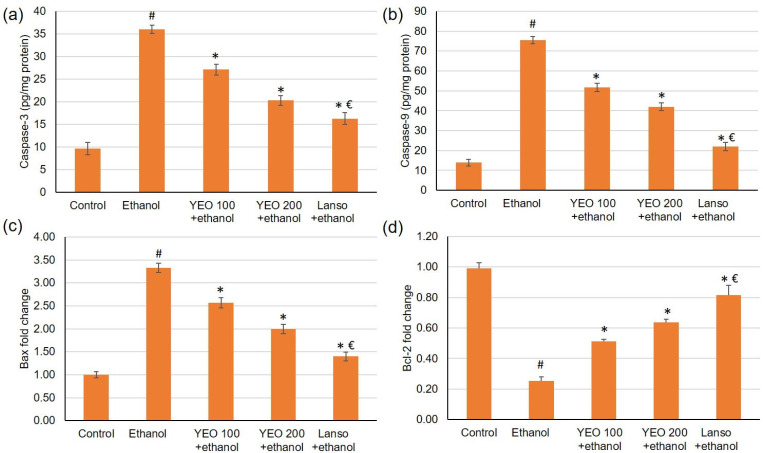
Effects of YEO (100 and 200 mg/kg) administration in acute gastric mucosal injuries induced via ethanol in rats on apoptotic indicators including (**a**) caspase-3, (**b**) caspase-3 and 9, and the gene expression of (**c**) Bax and (**d**) Bcl2. All data are quantified as mean ± SE, (*n = 6*). (#) defines statistically significant related to the control, (*) signifies statistically significant related to the ethanol-induced gastric mucosal injuries, and (€) designates statistically significant related to YEO 200 + ethanol via one-way ANOVA afterwards Tukey’s post hoc test (*p* < 0.05).

The gene expression of Bcl-2 was reduced, while Bax gene expression was elevated in the ulcer group when related to control animals. Upon the pretreatment with YEO (100 and 200 mg/kg) and lansoprazole, the gene expression of Bcl-2 was up-regulated, showing percentage changes of 102.63%, 151.68%, and 223.26%, respectively, related to the ethanol group, Figure 6. As for Bax gene expression, it was substantially lowered in groups pretreated with YEO and lansoprazole showing a percentage reduction of 22.86%, 39.98%, and 57.91%, respectively, when related to ethanol-induced gastric injuries, Figure 6. Regarding caspase-3 and 9, they were elevated in ulcerated animals, but in the groups pretreated with YEO or lansoprazole, caspase-3 and 9 were significantly lowered as demonstrated in Figure 6.

## 3. Discussion

Uncontrolled consumption of alcohol and spirits has increased the risk of gastric ulcers all over the world [25]. Extreme alcohol ingestion causes injury to the stomach by damaging the mucosal barrier integrity [26]. Gastric ulcers are caused by the reduction in the mucus-covering layer of the stomach, as a consequence of excess acid or pepsin secretion. Many factors may contribute to this corrosive development, such as infection with *Helicobacter pylori*, smoking, the misuse of alcohol, or the prolonged use of NSAIDs [27]. Many plants’ essential oils or their components demonstrated very promising activities against various types of ulcers and could be looked at as potential antiulcer agents [28,29,30].

The existing investigation examined the gastroprotective actions of YEO against ethanol-induced gastric ulcers in rats. Oral ingestion of ethanol caused numerous macroscopic alterations as well as microscopic alterations, confirming the injurious actions of ethanol. Ethanol prompts gastric lesions as it infiltrates and digests the gastric wall with its proteolytic and hydrolytic activities [31]. Earlier studies showed several microscopic and macroscopic modifications associated with the ingestion of ethanol [5,32,33]. Furthermore, ethanol-administered animals exhibited amplified gastric ulcer surface area and gastric volume accompanied by a substantial reduction in Alcian blue binding capacity to the mucosa, pH, and pepsin activity of gastric juice. Amplified hydrogen ion concentration is a destructive element facilitating gastric damage; thus, the gastric pH of ethanol-administered rats was reduced, as revealed by the current study and by previous studies as well [34]. Animals pretreated with YEO alleviated the ethanol-induced microscopic and macroscopic alteration, contributing to the preservation of the gastric wall structure. In addition, animals pretreated with YEO displayed reduced ulcer surface and gastric volume, complemented by an increase in the Alcian blue binding ability of the mucosa, pH value, and pepsin activity. These macroscopic and microscopic examinations, together with these outcomes, implied that YEO might ameliorate ethanol-induced gastric mucosal injuries.

One of the proposed mechanisms through which YEO may act is the Nrf2/HO-1 pathway. Transcription factor, nuclear factor-erythroid 2 related factor 2 (Nrf2) plays a crucial part in cell defensive mechanisms via maintaining the cell antioxidants ability [6], inhibiting pro-inflammatory signaling by reducing NF-κB [7]. The subsequential elements of Nrf2 signaling embrace the antioxidant enzymes HO-1, CAT, and GSH. It was reported that heme oxygenase-1 (HO-1) retains defensive qualities against ethyl alcohol-induced ulcers via the Nrf2/HO-1 pathway [8,35]. The immunohistochemical and gene expression results exposed that the ethanol displayed an escalated expression of the Nrf2 and HO-1. Furthermore, pretreatment with YEO causes further up-regulating in the expressions of Nrf2 and HO-1. A preceding study showed that yarrow oil suppressed the inflammatory responses of LPS-stimulated RAW 264.7 macrophages via the down-regulating of the HO-1 expression [36].

Extreme ingestion of ethanol provokes inflammation with the release of inflammatory cytokines as well as macrophages and lymphocytes infiltrated to the inflammation site [37,38]. The infiltrating of immune cells, especially neutrophils results in connexin destruction and mucosal barrier damage, which ultimately leads to gastritis [39]. Furthermore, these cytokines amplified oxygen-derived free radicals, thus aiding to the development of gastric ulcers [40,41]. Consistent with the preceding results, our results indicated that ethanol amplified the cytokines in the serum levels, including IL-1β, IL-6 and TNF-α. However, these pro-inflammatory cytokines levels were reduced by the pretreatment with YEO. Likewise, a previous study accomplished in our laboratory proved that YEO depressed ulcerative colitis via reducing of the inflammatory signs. It down-regulated NF-κB, TNF-α expression, IL-6 serum level as well as up-regulated peroxisome proliferator-activated receptor gamma (PPAR-γ), and transforming growth factor-β expression and restored IL-10 level [12]. In addition, YEO suppressed the inflammation associated with LPS-stimulated RAW 264.7 macrophages via the down-regulating the inducible iNOS, COX-2, TNF-α, IL-6 and HO-1 expression [36]. In addition, yarrow oil exhibited potent anti-inflammatory potential, especially in topical application [16].

PGE_2_ is one of the gastric mucosa defensive factors as it regulates gastric acid secretion, stabilizes the mast cell membrane, and stimulates the repairing measures, thus having a substantial part in the ulcer prevention as well as healing [3]. Therefore, PGE_2_ diminished level within the gastric mucosa results in ulceration and/or alleviation of the already existing ulcers [42,43]. Results of the current study disclosed that ethanol ingestion declined PGE_2_ level. Another gastric mucosa defensive factor is NO, which stimulates the mucus and bicarbonate synthesis, maintains the blood flow, and inhibits inflammation [44]. Consistent with previous reports [37,41], the results showed that serum and gastric NO were significantly reduced in ethanol-induced gastric ulcers, whereas pretreatment with YEO restored both serum PGE_2_ and serum and gastric NO levels.

An established relationship exists between oxidative stress during the development of ethanol-induced gastric ulcer [37]. ROS reacts with lipids to produce lipid peroxides causing extensive damage [45]. In the existing investigation, the amplified production of free radicals was associated with the membrane destruction detected in ethanol-induced gastric ulcers, as evidenced by the elevated serum and gastric lipid peroxidation (MDA). ROS are usually trapped by the natural antioxidant protective system, which compromises antioxidant enzymes, e.g., SOD and CAT [46]. Diminished GSH, SOD, and CAT enzyme activities contribute to ethanol-induced oxidative damage [47]. As revealed in the outcomes of the present study, ethanol exposure reduced GSH, SOD, and CAT activities. Conversely, YEO lowered the serum and gastric levels of MDA and restored the antioxidant enzyme GSH, SOD, and CAT activities in both gastric tissues and serum, identifying YEO’s antioxidant potential and further complying with the gastroprotective potential against ethanol-induced ulcers. Previously, YEO suppressed LPS-stimulated RAW 264.7 macrophage [36], via reducing NO and superoxide anion synthesis, lipid peroxidation, and glutathione content [36].

Ethanol-induced oxidative stress, together with inflammation, initiates the apoptosis intrinsic pathway resulting in activating Bax and deactivating Bcl2 [48,49]. The activated Bax translocates into the mitochondria, triggering the discharge of other pro-apoptotic elements to the cytoplasm, which initiates caspase 3, resulting in cell death [50,51]. Results showed that the gene expression of Bcl-2 was reduced, whereas Bax gene expression and caspase-3 and 9 were both elevated in the ulcer group. In contrast, pretreatment with YEO amplified the gene expression of Bcl-2 and reduced Bax gene expression as well as caspase-3 and 9 levels indicating restraining the apoptosis process arising with ethanol administration.

The phytochemical components of YEO may vary quantitatively in the relative percentage of each component; however, the essential oil qualitatively processes almost the same components. The chemical composition of our YEO sample, used in the current study, was revealed in an earlier study [12] by our laboratory to contain 6 components as major; Germacrene D (26.15%), β-caryophyllene (10.35%), and chamazulene (10.04%), as the major sesquiterpene; sabinene (14.28%), β-pinene (11.40%), and borneol (5.26%), as major monoterpenes. These major components may contribute, alone, or in combination, to the protective effect of YEO, discovered in the hereby study. These components may exert this protective effect through their antioxidant, anti-inflammatory, or antiapoptotic action. For example, chamazulene is recognized for its anti-inflammatory ability [52], and so is β-caryophyllene [53] and Germacrene D [54]. β-caryophyllene and germacrene D diminished the concentrations of the inflammatory mediators IL-1β, IL-6, and TNF-α, in LPS-stimulated cells [55]. Both Sabinene [56] and Borneol [57] inhibited the production of NO and lowered the levels of the inflammatory markers, including NO, TNF-α, and IL-6.

## 4. Materials and Methods

### 4.1. Plant Materials, and YEO Isolation

The plant materials’ collection and manipulation were described, as previously published by our laboratory [12]. Briefly, the whole yarrow plant (*Achillea millefolium L.*, family Asteraceae) was collected, identified, dried, and exposed to hydro-distillation via Clevenger-type apparatus for 3 h. The volatile oil fraction was recovered and dried over anhydrous sodium sulfate and stored at 4° C until use. The obtained YEO was analyzed using gas chromatography, and the results were previously published in [12].

### 4.2. Experimental Animals and Ethical Issues

King Faisal University Research Ethics Committee (KFU-REC) granted its ethical authorization with approval number KFU-REC-2022-APR-EA000566. The study procedure was permitted by KFU-REC, which approved all the procedures. Healthy male adult Wistar rats were included in this experiment. Rats were retained under optimal standardized laboratory conditions at 25 ± 2 °C in a 12-h light-dark cycle at the College of Medicine animal house, King Faisal University.

### 4.3. Experimental Design

Wistar rats (aged 6–8 weeks and weighing 200–220 g) were distributed randomly into five groups (*n = 6*), and the experiment was accomplished as mentioned earlier [33]. Group 1 (control group), in which rats were given 10% Tween 20 (the vehicle) orally for two weeks. Group 2, or ethanol group, in which rats were given absolute ethanol (5 mL/kg) orally to produce peptic ulcerated animals on the last day of the experiment. YEO 100 and 200 mg/kg + ethanol (Group 3 and 4) in which animals were pretreated with YEO 100 and 200 mg/kg, respectively, orally dissolved in 10% Tween 20, for two weeks preceding the inauguration of gastric ulcer via ethanol. Group 5 (Lanso + ethanol) represents the reference control group, in which animals were pretreated with 20 mg/kg lansoprazole dissolved 10% Tween 20 orally for two weeks preceding the inauguration of gastric ulcer via ethanol. The doses of YEO were chosen depending on a study that was implemented in our laboratory [12]. A preliminary study was done to investigate the therapeutic doses of YEO in experimental animals, using concentrations from 1 to 500 mg/kg, from which these two doses were chosen to continue the investigation.

### 4.4. Induction of Gastric Ulcer

Former to the ethanol administration, animals were fasted for 24 h, with free access to drinking water until 2 h before ethanol administration. Gastric ulcer was prompted by absolute ethanol (5 mL/kg) using oral gavage. Four hours after absolute ethanol administration, animals were anesthetized using pentobarbital sodium (35 mg/kg, i.p.). Blood was collected from the abdominal aorta, and centrifuged at 4000 rpm for 15 min to acquire serum which was stored at −80 °C. Then, the rats were sacrificed, the stomachs were separated and drained into centrifuge tubes to measure gastric section indices. Stomachs were washed with ice-cold saline for macroscopic examination, gastric barrier mucus quantitative estimation, and to prepare stomach homogenate for subsequent analysis.

### 4.5. Gastric Mucosal Lesion Macroscopic Examination

Gastric macroscopic injury was photographed and evaluated by blinded histopathologist and recorded as mentioned before [58]. The macroscopic gastric damage score used was as follows: 0 indicating zero lesions, 1–2 signifying small lesions, 3–4 signifying small ulcer, 5–6 indicating large ulcer, and 7 representing full of ulcers. The ulcer’s length and width were assessed using a planimeter (10 × 10 mm^2^) using a dissecting microscope. The gastric ulcerated area was assessed via counting the sum of small squares underlying the ulcer area. The ulcer area (UA) and the inhibition percentage (I %) were estimated as mentioned before [32,33] using the formulas:

Ulcer area UA (mm^2^) = number of small squares × 4 × 1.8.  The inhibition percentage (I%) =  ((ulcer area of control − ulcer area of treated)/ulcer area of control) × 100

### 4.6. Assessment of Gastric Indices

The stomach contents were drained into centrifuge tubes for volume and pH determination. The stomach content drain was centrifuged (4000 rpm, 10 min), and the obtained supernatant pH was measured using a digital pH meter. In addition, pepsin activity was estimated using the stomach secretion.

### 4.7. Gastric Barrier Mucus Quantitative Estimation

The gastric wall mucus was assessed using Alcian blue, as mentioned before [59]. The stomach’s glandular part was weighed and mixed with 10 mL of 0.1% *w/v* Alcian blue solution for 2 h, and the extra color was removed by washing the solution two times using 0.25 M sucrose. Alcian blue dye mixed with gastric wall mucus was extracted via irregular shaking for 1 min every 30 min for 2 h with 0.5 M magnesium chloride (MgCl_2_). Diethyl ether is added to the blue concentrate to obtain an emulsion which was centrifuged at 3000× *g* for 10 min. The fluid layer absorbance was assessed at 580 nm. The Alcian blue extent for every gm of wet glandular tissue was, afterward, calculated.

### 4.8. Histopathological and Immunohistochemistry (IHC) Microscopically Examinations

The gastric tissues were fixed with 10% (*v/v*) formalin and paraffin-embedded to obtain paraffin-embedded gastric tissue sections, which will be used in histopathological and IHC evaluations.

Gastric sections (5 µm) were stained using hematoxylin and eosin (H&E) for histological assessment. The pathological fluctuations were recognized using a microscope by a blinded histopathologist. The hemorrhagic damage, epithelial cell loss, and edema with leucocytes were recorded, and the lesions’ extent was categorized from 1 to 5 according to the severity. One indicated the minimal degree of lesion (less than 1%), two signified slight (1–25%), three demonstrated moderate (26–50%) degree of lesion, four implied moderate/severe (51–75%), and five showed severe/high (76–100%) degree of lesion.

For the IHC procedure, the expression of Nrf2 and HO-1 were determined. Briefly, stomach sections were blocked using 3% H_2_O_2_ in methanol (21–25 °C, 30 min) and washed with phosphate-buffered saline three times. The gastric sections were incubated with Nrf2 and HO-1 antibodies (1:100, Thermo Fisher Scientific) overnight at 4 °C, followed by goat anti-rabbit-horseradish peroxidase (HRP) conjugated IgG antibody for 1 h at 37 °C. The gastric sections were settled with 1% diaminobenzidine, counterstained with 1% hematoxylin, and mounted. The gastric sections were assessed using a microscope built-in with a digital camera. The area of the IHC reaction was chosen, and then the average optical density in the designated area was assessed. Positive cells were calculated with ×400 magnification, spotting 10 sequential fields for each animal. NIS-Elements software was utilized for quantitative analysis.

### 4.9. Gastric Homogenate Preparation

The gastric tissue was divided into small parts, homogenized by a Teflon homogenizer and centrifuged (4500 rpm, 15 min, 4 °C) to obtain supernatant. The obtained supernatant was used for the oxidative stress, antioxidant enzymes, caspase 3, and caspase 9 measurements. The entire experiment was performed at 4 °C.

### 4.10. Gene Expression Experiments (Real-Time PCR)

Real-time PCR was performed according to the technique used in our laboratory and described elsewhere [60]. Quantification analyses were completed via Opticon-2 Real-Time PCR reactor. Step PE Applied Biosystems analyzed real-time PCR results. Expression of the target genes were assessed and related to the reference gene (β-actin). β-actin expression was used for sample normalization, where the 2^−ΔΔCT^ equation was used for relative expression determination. The primers used are as follows: Nrf2 (NM_031789.2) F: 5′-CATTTGTAGATGACCATGAGTCGC-3′, R: 3′-ATCAGGGGTGGTGAAGACTG-5′; HO-1 (NM_ 012580.2) F: 5′-GTGCACATCCGTGCAGAGAA-3′, R: 3′-GTGCACATCC GTGCAGAGA A-5′; Bcl-2 (NM_016993.1) F: 5′-CCGGGAGATCGTGATGAAGT -3′, R: 3′-ATCCCAGCCTCCGTTATCCT-5′; Bax (NM_017059.2) F: 5′-GTGGTTGCCCTCTTCTAC TTTG-3′, R: 3′-CACAAAGATGGTCACTGTCTGC-5′; β-actin (NM_0 3144.3) F: 5′-TGACAGGATGCAG AAG GAGA-3′, R: 3′-TA GAGCCACCA ATCCACACA-5′.

### 4.11. The Assessment of Gastric Mucosa Defensive Factors

Serum levels of prostaglandin E_2_ (PGE_2_, ab287802) and serum and gastric levels of nitric oxide (NO, ab65328) were measured using ELISA kits that were acquired from Abcam Co., Eugene, OR, USA.

### 4.12. The Assessment of the Inflammatory Cytokines Markers

Serum levels of inflammation cytokines markers, comprising TNF-α (ab46070), IL-1β (ab100768), and IL-6 (ab100772), were evaluated using ELISA kits, which were obtained from Abcam Co., Eugene, OR, USA.

### 4.13. The Assessment of Oxidative Stress Status and Antioxidant Enzymes Activities

Serum and gastric levels of malondialdehyde (MDA; ab238537) were measured using an ELISA kit acquired from Abcam Co., Eugene, OR, USA. The serum and gastric levels of reduced glutathione (GSH; MBS706914), superoxide dismutase (SOD; MBS036924), and catalase (MBS726781) were assessed using ELISA kits, obtained from My BioSource (San Diego, CA, USA).

### 4.14. The Assessment of Apoptotic Markers

Cleaved caspase-3 (KHO1091) was obtained from Thermo Fisher Scientific Inc., Waltham, MA, USA, whereas caspase-9 (LS-F4141) was attained from Biocompare, CA, USA.

### 4.15. Statistical Analysis

Data in the current study are displayed as mean ± SD. For numerous associations, one-way ANOVA followed by Tukey–Kramer as a post hoc test was accomplished. Level of probability of 0.05 or less was utilized as the level of significance (*p* < 0.05). All analyses were executed via Graph Pad software v. 8.

## 5. Conclusions

The current study presented that YEO exerted a considerable gastroprotective action against ethanol-induced gastric lesions. This gastroprotective potential may be related to the enrichment of the antioxidant protective system and to deterring inflammation through decreasing IL-1β, IL-6, and TNF-α and detaining apoptosis via regulating Bax, Bcl-2, and caspase 3 and 9. YEO exhibited antioxidant, anti-inflammatory, and anti-apoptotic activities, possibly through instigating the Nrf2/HO-1-related pathway. This study adds to the pool of YEO pharmacological activities and reveals part of the underlying mechanism of action. This study could be implemented in an anti-alcoholism program through using YEO as a protective agent against misused-alcohol-induced gastric ulcers in patients at risk.

## Figures and Tables

**Table 1:** The actions of the YEO on gastric ulcer area, inhibition % and Alcian blue binding ability in various experimental groups.

Animal’s Group	Ulcer Area (mm^2^)	Inhibition (%)	mg Alcian Blue/g Tissue
Control	-	-	620.75 ± 15.23
Ethanol	735.8 ± 30.7 #	-	156.35 ± 10.41 #
YEO (100 mg/kg) + ethanol	360.49 ± 10.76 *	52.6% *	340.95 ± 13.72 *
YEO (200 mg/kg) + ethanol	241.56 ±13.68 *	64.28% *	448.93 ± 13.6 *
Lansoprazole + ethanol	192.45 ±15.32 *€	70.16% *€	571.8 ± 21.5 *€

All data are quantified as mean ± SE, (*n* = 6). (#) defines statistically significant related to the control, (*) signifies statistically significant related to the ethanol-induced gastric mucosal injuries and (€) designates statistically significant related to YEO 200 + ethanol via one-way ANOVA after Tukey’s post hoc test (*p* < 0.05).

**Table 2:** Effect of yarrow oil on gastric pH and volume and pepsin activity of gastric juice in various experimental groups.

Animal’s Group	Gastric pH	Gastric Volume (mL)	Pepsin Activity (U/mL)
Control	6.3 ± 1.59	0.14 ± 0.03	2857.50 ± 202.84
Ethanol	4.35 ± 0.47 #	2.85 ± 0.74 #	1046.33 ± 178.03 #
YEO (100 mg/kg) + ethanol	5.39 ± 2.23 *	2.01 ± 0.24 *	1352.00 ± 140.94 *
YEO (200 mg/kg) + ethanol	5.7 ± 2.43 *	1.81 ± 0.31 *	1727.76 ± 151.09 *
Lansoprazole + ethanol	6.01 ± 0.51 *€	1.27 ± 0.42 *€	2092.13 ± 145.82 *€

All data are quantified as mean ± SE, (*n* = 6). (#) defines statistically significant related to the control, (*) signifies statistically significant related to the ethanol-induced gastric mucosal injuries, and (€) designates statistically significant related to YEO 200 + ethanol via one-way ANOVA after Tukey’s post hoc test (*p* < 0.05).

## Data Availability

Not applicable.
